# Treatment of Periostitis

**Published:** 1878-11

**Authors:** A. M. Holmes

**Affiliations:** Morrisville, N. Y.


### ARTICLE VIII.

### *Treatment of Periostitis.*

BY A. M. HOLMES, MORRISVILLE, N. Y.

I beg leave to call attention to the use of Gelseminum as an efficient remedy in the treatment of periostitis and other painful diseases of the teeth and surrounding tissues.

As a remedy for neuralgia and kindred diseases of 5th pair of nerves, it seems as nearly a specific as medical science provides for the cure of any disease.

I can refer to my personal experience in its use for treatment of that exceedingly painful affection of the face, combining the characteristics of neuralgia and rheumatism, called by Drs. Inman and Anstie, myalgia, resulting in prompt and absolute relief.

But my object now is to refer more particularly to its efficacy in the treatment of periostitis and other painful conditions of the teeth and surrounding tissues, with the assurance that my experience in its use has been far more satisfactory and successful than with the usual treatment adopted in such cases.

I recently prescribed this remedy in a case possessing the characteristic symptoms of exostosis of the root of an incisor, which had resisted other constitutional as well as local treatment. The remedy was taken in sufficient quantity to relieve the excruciating pain with which the patient was suffering at the time—requiring about 30 drops of Tilden & Co.'s extract of Jessamine in 5-drop doses half-hourly—which was followed with twelve to fifteen drops daily, on retiring, for four days, when all soreness had disappeared from the alveolus (the first time in months,) and the tooth has remained comparatively comfortable since, now more than 12 months.

Gelseminum (or yellow Jessamine) being a powerful nervous and arterial sedative, would seem to indicate it as an efficient remedy in the treatment of inflamed or sensitive dentine, where constitutional remedies are called into requisition, and in particular to prevent the dangerous effects of congestion in the pulps of teeth after resorting to capping.

My experience in the use of this remedy for the purposes indicated has proved so satisfactory to myself, and gratifying to my patients, that I take pleasure in calling attention to its use. As there is so great a difference in the extracts or preparations, and the remedy acts powerfully on the nervous system, care should be exercised in its use, and an exact knowledge of the strength of the preparation prescribed, as one drop of the stronger extracts often proves a sufficient dose.—*Dental Advertiser.*

---

